# A Case of Self-Inflicted Penile Ulceration

**Published:** 1989-11

**Authors:** D. S. Allen

**Affiliations:** Psychiatric Division, Basingstoke District Hospital


### A Case of Self Inflicted Penile Ulceration

**D. S. Allen**
Psychiatric Division, Basingstoke District Hospital

### INTRODUCTION

It is difficult to make a confident diagnosis of self-inflicted injury although certain criteria have been established. It can be time consuming to collect the necessary evidence and medical and nursing staff often become frustrated by the patient's lack of 'willingness to get better'. Finally, there has been little success in treating these patients.

### CASE REPORT

A 23 year old fork lift truck driver presented with a 6 week history of a painful, red and swollen foreskin. He was found to have a phimosis with a sub-preputial discharge. Culture of the latter and a urethral smear were both negative as was his serology. He was treated with Metronidazole and Natamycin. There was no change over the next month and a circumcision was performed. An indurated mass was found ventrally at the base of the glans. Pus from this grew Bacteroides and he was given a further course of Metronidazole. Histology showed a granulomatous reaction.

When he was seen a month later there was a granulomatous area near the frenulum and 2 ulcers on the dorsum of the coronal sulcus where the suture line had parted. Tests for T.B. and sarcoid were negative and he was treated with Erythromycin. 5 weeks after this the dorsal ulcers had healed, but the granulomatous area had developed into an ulcer which had extended to undermine the normal skin. Over the next 2 months this increased in size until it measured 2 × 3 cm, although parts of it did heal.

At this stage he was re-admitted to hospital and Dextranomer was applied to the ulcer daily. A secondary infection with *Staph. Aureus* was treated with Flucloxacillin. A biopsy showed a non specific acute ulcer with inflammation of the adjacent tissues. Tests done previously were negative on repetition and an electrophoretic strip showed only signs of chronic non-specific inflammation. A modified glucose tolerance test was normal.

By this stage there was a strong suspicion amongst all the staff that the ulcer might be self inflicted. This was based on the absence of an obvious cause or pathological perpetuating factors and the patient's seeming indifference to his problem. A psychiatric history did not reveal any obvious psychodynamic factors which might have explained his behaviour other than the fact that at about the time that the original ulcers had healed, he had met a woman to whom he had later become engaged. During the time that they were courting there was no possibility of sexual intercourse being 'considered' because of the ulcer. The patient professed not to be concerned about this.

A further 3 weeks of simple dressings led to some improvement but on return from a subsequent period of leave the lesion had deteriorated significantly and the patient was enclosed in a plaster cast encompassing his penis and attached to this trunk by a 'figure of eight'. Unfortunately this slipped off several times and had to be abandoned, but during the time that it was in situ there was noticeable healing of the ulcer.

At this stage the putative diagnosis was discussed with the patient for the first time. He remained calm, but consistently denied injuring himself. It was noticed by the nursing staff that he tended to dominate the ward, keeping the television in his room, only speaking to people when he chose and shouting at new nurses who 'did not know how to do the dressing properly'. At a further interview he was asked questions to try and elicit a motive for his supposed actions. One question concerned the possibility of his having homosexual feelings. Afterwards he complained bitterly about this question having been posed.

Eight months after the original presentation the ulcer was 6 cm long and encompassed two thirds of his penis. As he had not benefited from in patient treatment, he was discharged. He did not attend for follow up but it was learnt that the ulcer had eventually healed some months after discharge.

### LITERATURE ON SELF INJURY

The literature on self injury deals largely with discussion of the 'wrist cutting syndrome' and associated phenomena (1, 2, 3). References to genital mutilation in males are usually to cases of auto-amputation and castration (4, 5, 6, 7), although genital mutilation of a less dramatic nature in females has been reported by several authors (8, 9, 10). In some cases mutilation has resulted from pushing objects up the urethra for sexual stimulation (4). In one case a patient had a compulsion to dig and scratch at his genitalia but he admitted to this freely, which, although not unusual in cases of wrist cutting, is atypical of other types of refractory skin lesions (2, 11).

### MOTIVES

Motives are sometimes obvious, as in cases of attempted abortion. However reasons for self injury are often unclear and may even be performed subconsciously. It may bring positive benefit in the form of attention and sympathy from friends and doctors, or it may bring negative benefit if hospital admission takes the person away from the pressures at home or work. In some it may be a means of reducing guilt (of sexual desires for example); others may have a need for sensory stimulation (12). A small minority are due to an aberrant physiological process like Lesch Nyhan Syndrome (13).

Menninger believed self injury to be a form of 'focal suicide' (14). Excluding religious or customary forms and organic disorders, he described 2 types of 'mutilator': the neurotic performs actions knowingly, by a compulsion which he is unable to control, and the psychotic injures himself subconsciously. In both cases the motive is often punishment, stopping short of the ultimate sanction (suicide); the reason for the person wanting to punish himself in many cases is guilt, which often has sexual connotations. Although parts other than the genitalia are usually mutilated, especially in neurotics, there is sometimes a sexual symbolism to that part in the person's experience.

Reasons for genital mutilation are probably more complex. In a study of a series of cases of auto-amputation and castration, several factors were thought to contribute to the final act which was the culmination of many years of genital self mutilation, often starting in childhood (5). These include a severely impoverished childhood with an over-controlling mother and a father absent either physically, or by virtue of his weakness of character. These men have usually been intensely sexually confused over a long period of time and have submissive, masochistic relationships with women; they usually have strong female identification. They often see genital mutilation as the only way of relieving the depression they feel.

Fisch summarizes the main dynamic factors predisposing to genital self mutilation in males as guilt, rage, fear and loss connected with sexuality, homosexuality, rejection of masculinity, 'femaleness' and fantasies concerning incest or birth (7).

### DIAGNOSIS

Self injury, like hysteria, is always at the bottom of any differential diagnosis list. Even when every other possibility has been excluded it is often very difficult to find positive proof in the face of the patient's denial. Based on a series of non-healing ulcers and other lesions, criteria have been described which provide a framework for diagnosis (11):

1. The atypical nature of the lesion (e.g. ulcers spreading in an inexplicable manner).
2. The frequency with which such lesions seem to arise in existing wounds.
3. A previous history of similar lesions.
4. No indignation at any time when the patient is accused of causing the lesion, although this is denied.
5. No replies actually convince the doctor that his diagnosis is wrong.

Catching the patient 'in the act' often convinces the doctor but this is not always conclusive proof and can quite easily be argued away by the patient.

### TREATMENT

Despite the extensive literature on self injury little has been written on treatment. Not surprisingly, preventing the patient from touching the lesion, if this is possible, effects a temporary cure and virtually proves the diagnosis. However as the underlying problem is a psychiatric one, it is likely to recur, occasionally at a different site.

Patients often cause strong feelings in staff who end up arguing amongst themselves. By keeping the external world in polarity and conflict, the patient resists exploration of himself with all its attendant anxiety. Therefore it is important to unify splits in opinion amongst staff in discussion with the patient, to prevent him from believing that they are united against him (15).

Management can be divided into 3 phases (16): In the early (admission) phase the priorities are diagnostic evaluation, reduction of symptoms and facilitation of compliance with any surgical procedures. In the middle (post-surgical) phase it is important to allow the patient and his family to ventilate their feelings and to provide explanations of surgical procedures in order to reduce anxiety and guilt. The patient's suitability for psychotherapy can also be explored at this stage and begun if appropriate. The late (approaching discharge) phase is concerned with formulating clear future plans, which should include family support, and tackling psychodynamic issues.

### REFERENCES

1. PAI, P. N. (1969) The syndrome of delicate self-cutting. *British Journal of Medical Psychology* **42**, 195–206.
2. LYELL, A. (1972) Dermatitis artefacta and self-inflicted disease. *Scottish Medical Journal* **17**, 187–199.
3. SIMPSON, M. A. (1976) Self-mutilation. *British Journal of Hospital Medicine* **October 1976**, 430–438.
4. BEILIN, L. M. (1953) Genital self-mutilation by mental patients. *Journal of Urology* **70**, 648–655.
5. BLACKER, K. H., WONG, N. (1963) Four cases of autocastration. *Archives of General Psychiatry* **8**, 169–176.
6. MENDEZ, R., KIELY, W. F., MORROW, J. W. (1972) Self-emasculation. *Journal of Urology* **107**, 981–985.
7. FISCH, R. Z. (1987) Genital self-mutilation in males: Psychodynamic anatomy of a psychosis. *American Journal of Psychotherapy* **41(3)**, 453–458.
8. GOLDFIELD, M. D., GLICK, I. R. (1970) Self-mutilation of the female genitalia. A case report. *Diseases of the Nervous System* **31**, 843–845.
9. FRENCH, A. P., NELSON, H. L. (1972) Genital self-mutilation in women. *Archives of General Psychiatry* **27**, 618–620.
10. SIMPSON, M. A. (1973) Female genital self-mutilation. *Archives of General Psychiatry* **29**, 508–510.
11. BATTLE, R. J. V., POLLITT, J. D. (1964) Self-inflicted injuries. *British Journal of Plastic Surgery* **17**, 400–412.
12. CARR, E. G. (1977) The motivation of self-injurious behaviour – a review of some hypotheses. *Psychological Bulletin* **84(4)**, 800–816.
13. LESCH, M., NYHAN, W. I. (1964) A familial disorder of uric acid metabolism. *American Journal of Medicine* **36**, 561–571.
14. MENNINGER, K. (1938) In: *Man Against Himself*. Harcourt Brace and World. New York. 203–250.
15. PODVOLL, E. M. (1969) Self-mutilation within a hospital setting – a study of identity and social compliance. *British Journal of Medical Psychology* **42**, 213–221.
16. YOUNG, L. D., FEINSILVER, D. L. (1986) Male genital self-mutilation: Combined surgical and psychiatric care. *Psychosomatics* **27(7)**, 513–517.

